# Mixed methods evaluation explains bypassing of vouchers in micronutrient powder trial in Mozambique

**DOI:** 10.1111/mcn.12718

**Published:** 2019-10-17

**Authors:** Alison Tumilowicz, Marieke Vossenaar, Kirstine Kjaer, Julia Vettersand, Edna Possolo, Gretel H. Pelto, Maria Elena Jefferds, Augusto Norte, Katia dos Santos Dias, Nadia Osman, Wendy Gonzalez, Alia Poonawala, Lynnette M. Neufeld

**Affiliations:** ^1^ Global Alliance for Improved Nutrition (GAIN) Geneva Switzerland; ^2^ Independent Consultant Geneva Switzerland; ^3^ Independent Consultant Maputo Mozambique; ^4^ Department of Nutrition Ministry of Health Maputo Mozambique; ^5^ Division of Nutritional Sciences Cornell University Ithaca New York; ^6^ Division of Nutrition, Physical Activity, and Obesity Centers for Disease Control and Prevention (CDC) Atlanta Georgia; ^7^ A‐Consultants Statistical Services Mendoza Argentina

**Keywords:** ethnography, evaluation, infants and young nutrition, micronutrients, mixed methods, Mozambique

## Abstract

Micronutrient powders (MNP) are recommended by the World Health Organization as an effective intervention to address anaemia in children. A formative process evaluation was conducted to assess the viability of a model using free vouchers in two districts of Mozambique to deliver MNP and motivate adherence to recommendations regarding its use. The evaluation consisted of (a) an examination of programme outcomes using a cross‐sectional survey among caregivers of children 6–23 months (*n* = 1,028) and (b) an ethnographic study to investigate delivery experiences and MNP use from caregiver perspectives (*n* = 59), programme managers (*n* = 17), and programme implementers (*n* = 168). Using a mixed methods approach allowed exploration of unexpected programme outcomes and triangulation of findings. The survey revealed that receiving a voucher was the main implementation bottleneck. Although few caregivers received vouchers (11.5%, CI [9.7, 13.6]), one‐fourth received MNP by bypassing the voucher system (26.3%, CI [23.6, 29.0]). Caregivers' narratives indicated that caregivers were motivated to redeem vouchers but encountered obstacles, including not knowing where or how to redeem them or finding MNP were not available at the shop. Observing these challenges, many programme implementers redeemed vouchers and distributed MNP to caregivers. Virtually, all caregivers who received MNP reported ever feeding it to their child. This study's findings are consistent with other studies across a range of contexts suggesting that although programmes are generally effective in motivating initial use, more attention is required to improve access to MNP and support continued use.

**Key messages**

- The novelty and complexity of the voucher system impeded delivery of micronutrient powders (MNP) to caregivers.
- Dedicated and resourceful programme implementers found strategies to overcome challenges, primarily through eliminating the need for caregivers to use vouchers.
- Most caregivers who received MNP initiated feeding it to their child, a much smaller proportion continued to use MNP.
- Findings point to the importance of supporting frontline workers in communities to carry out nutrition promotion activities.
- The results of this study supported by experiences elsewhere suggest that programmes are generally effective in motivating initial MNP use; more attention is required to improve access to MNP and support continued use.

## 1 INTRODUCTION

Micronutrient powders (MNP), a mixture of vitamins and minerals, enclosed in single‐dose sachets that are stirred into a child's portion of food immediately before consumption, are one of the few World Health Organization (WHO) recommended nutrition interventions to address anaemia and iron deficiency in young children and improve the quality of complementary feeding of young children 6–23 months (De‐Regil, Suchdev, Vist, Walleser, & Peña‐Rosas, 2013; World Health Organization [WHO], 2016). MNP interventions have been implemented in a wide range of countries using different combinations of delivery models, platforms, and channels, illustrating their potential for MNP distribution (Reerink et al., 2017). However, there is insufficient evidence to guide effective, context‐specific delivery of the intervention in different systems, cultures, and communities (Reerink et al., 2017). More investigations to examine implementation are needed in order to understand what happened in a programme and how that could affect programme outcomes or impacts (Vossenaar et al., 2017).

In Mozambique, two third (68.7%) of children 6 months to 5 years suffer from anaemia (Ministerio da Saude [MISAU] et al., 2011). The Ministry of Health (MISAU, acronym in Portuguese) developed in 2010 a comprehensive action plan (Government of the Republic of Mozambique, 2010), detailing a policy related to a number of nutrition interventions. Within the context of counselling to improve infant and young child (IYC) feeding practices and aligned with recent WHO guidance on the use of MNP for point‐of‐use fortification of foods consumed by IYC (WHO, 2016), the government recommended the inclusion of MNP as part of the national strategy. Many development partners work closely with MISAU, and several were asked to pilot‐test the delivery of MNP across different regions of the country to inform how MNP delivery could be scaled‐up in the country.

In two districts of Sofala Province, Global Alliance for Improved Nutrition (GAIN), Save the Children (SC), and Population Services International (PSI) supported MISAU to implement a trial using a voucher distribution system. Caregivers of children aged 6–23 months were provided with IYC feeding counselling as part of ongoing MISAU and SC programmes and vouchers that could be redeemed without cost for MNP called *VitaMais* at vendors of PSI's existing sales platform. The logic behind the design decision was that using a market‐based platform would alleviate the burden on the public health supply system while increasing accessibility of MNP through distribution points located in communities where caregivers live. Testing the use of vouchers was also relevant to inform the feasibility of using them to subsidize MNP cost (rather than provide MNP free of cost upon redemption).

**Figure 1:**
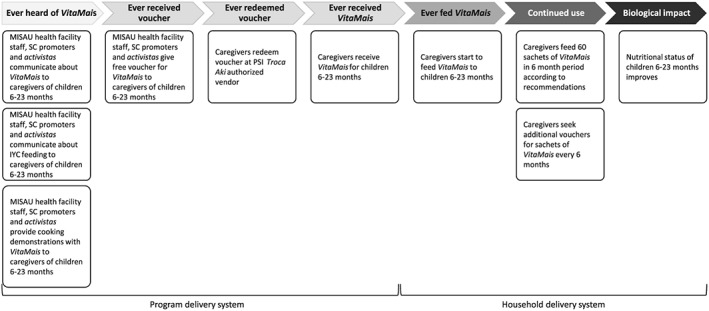
Programme impact pathway for micronutrient powder delivery system using vouchers in Mozambique. *VitaMais*: brand of micronutrient powder used in the programme; MISAU: Mozambique Ministry of Health; SC: Save the Children; *activistas*: volunteer community health workers; IYC: infant and young child; *Troca Aki* (translated “Exchange Here”): local commercial shops that were registered with Population Services International

A formative process evaluation was conducted by GAIN with technical assistance from the Division of Nutrition, Physical Activity, and Obesity, Centers for Disease Control and Prevention to assess the viability of the specific model implemented in Mozambique to deliver MNP and motivate adherence to recommendations regarding its use. To illustrate how the delivery of the programme would result in continuing and appropriate use of MNP, we developed a programme impact pathway (PIP; Habicht & Pelto, 2012) through consultation with programme staff and an extensive literature review on factors affecting MNP adherence (Tumilowicz, Schnefke, Neufeld, & Pelto, 2017). The PIP is composed of two systems: (a) a programme delivery system including the processes in which the intervention is delivered to the household and (b) a household delivery system that consists of all steps needed for a child to consume a biologically impactful dose. Figure 1 presents the flow of the PIP in the form of programme component exposures and actions by the caregiver. The PIP describes the transmission of MNP and behaviour change communication (BCC) activities from the programme delivery system to the household delivery system and the sequence of activities to prepare food with MNP and feed it to the child.

We identified two main questions to be addressed in the evaluation: (a) What proportion of caregivers received the intervention and adhered to MNP recommendations, and (b) how did delivery and utilization processes affect programme outcomes, including coverage and utilization of MNP. Examination of these two questions required a complex study design and mixed methods. Therefore, we created two streams of inquiry: (a) an examination of programme outcomes using cross‐sectional survey data and (b) a focused ethnographic study (FES) to investigate delivery experiences and MNP use from perspectives of caregivers, programme managers, and implementers (Cove & Pelto, 2010; Pelto, Armar‐Klemesu, Siekmann, & Schofield, 2013). The central purpose of ethnography is to obtain the emic view—the insider's perspective—that allows for the discovery of conditions and behaviours that are not foreseen (Pelto & Pelto, 1978). The difference between ethnography and other social science methods of investigation is that ethnographers aim to discover what people do and why, then use what is learned to build theories rather than assess the validity of preconceived theories (Schensul & LeCompte, 2012). In this paper, we focus on what we unexpectedly learned through the mixed methods formative evaluation design about the delivery and redemption of vouchers and how and why programme delivery unfolded as it did primarily from the ethnographic study results.

## 2 METHODS

### 2.1 Description of programme delivery system

The trial aimed to deliver one voucher every 6 months redeemable for 60 sachets of MNP to approximately 20,000 children 6–23 months, equivalent to about 30% of all children of eligible age from January 2016 to March 2017 in Beira and Dondo districts of Sofala Province (Instituto Nacional de Estatística, 2013). In 2013, the estimated population of Beira was 456,005 (Instituto Nacional de Estatística, 2013). The capital of the province is in Beira, and most of the district is considered urban or peri‐urban. The estimated population of Dondo was 161,752 (Instituto Nacional de Estatística, 2013). Dondo is adjacent to Beira, and the main highway from the coast to Zimbabwe transects the district. Along the highway, communities are peri‐urban, but a majority of the population resides in rural areas. MNP are locally branded as *VitaMais* (produced by Piramal, India) and contain 15 vitamins and minerals including iron and zinc at ~1× the recommended nutrient intake (FAO/WHO, 2004).

MISAU with the support of partners integrated instructions on MNP use into existing national IYC feeding materials, which were originally adapted from UNICEF's Community‐IYC Feeding Counselling Package (UNICEF, 2013). Using these materials and additional protocols how to distribute and redeem vouchers, GAIN trained “master trainers” from MISAU, SC, and PSI, who subsequently trained programme implementers during week‐long sessions in the districts.

Free vouchers for *VitaMais* were distributed at health centres and in communities to caregivers of children 6–23 months. In Beira, vouchers were distributed to caregivers by health workers, promoters, and community nutrition volunteers, called *activistas* in Portuguese. The promoters and *activistas* in Beira were recruited by SC to support the *VitaMais* trial. The SC promoters were paid a minimum monthly salary and worked at the health centre, whereas SC *activistas* were volunteers and worked in communities. SC promoters worked alongside District Directorate of Health of Beira City (DDS acronym in Portuguese) health workers to distribute vouchers for *VitaMais*. SC promoters also provided interpersonal counselling on *VitaMais* and IYC feeding, gave group education sessions (*palestras*), and cooking demonstrations to caregivers at the health centre. Supervised by SC promoters, SC *activistas* conducted the same activities at the community level.

In Dondo, implementation was solely conducted by the District Services of Health, Women and Social Action (SDSMAS, acronym in Portuguese) without support from SC. Vouchers and BCC activities (i.e., interpersonal counselling, *palestras*, and cooking demonstrations) were delivered by health workers in health centres and SDSMAS *activistas* (unpaid volunteers) at the community level. The SDSMAS *activistas* were already actively supporting health services, and the tasks related to the MNP trial were added to their existing activities. There were far fewer SDSMAS *activistas* active at the community level compared with SC *activistas* in Beira.

In both districts, caregivers were instructed to feed food mixed with one sachet of *VitaMais* to the child 6–23 months every day for 2 months and to return to the health centre or *activista* for a new supply after 6 months. BCC activities consisted of interpersonal counselling, *palestras*, and cooking demonstrations; no mass media communication or community activations activities took place.

The only MNP distribution in Beira and Dondo was through distribution of free vouchers redeemable for *VitaMais*. Caregivers could redeem vouchers in local commercial shops that were registered with PSI and identified with the brand *Troca Aki* (translated “Exchange Here”). PSI registered 44 *Troca Aki* vendors in Beira and 21 in Dondo, predominantly located near health centres and to a lesser extent in outlying communities. Upon redemption of the voucher, each caregiver was provided with three boxes containing 20 *VitaMais* sachets. There were two types of vouchers distributed (by the same system previously described): paper and electronic. The paper vouchers were physically given to the caregivers. The electronic vouchers were sent using SMS when the caregiver sent a code to the PSI electronic platform, called *Movercado*. The *Troca Aki* vendor received payment after sending the serial number of the paper or electronic voucher to the *Movercado* platform, which indicated that boxes of *VitaMais* had been distributed. *VitaMais* was the only available MNP in the districts and only available through voucher redemption at the *Troca Aki* shops. *Troca Aki* vendors were trained on the supply chain procedures and voucher redemption but received minimal orientation on recommendations regarding the appropriate use of *VitaMais*.

### 2.2 Ethical considerations

The study was approved by the MISAU Ethics Committee of Mozambique (Comité Nacional de Bioética para Saúde IRB00002657) on September 22, 2016 (Ref: 305/CNBS/16). Respondents provided written informed consent to participate in the study; if illiterate, a witness signature was obtained.

### 2.3 Cross‐sectional survey

The survey was conducted during January and February 2017, approximately 12 months after initiation of MNP distribution. A structured interview with precoded questions was used to collect data on MNP coverage and adherence outcomes, caregiver exposure to the elements of the programme delivery design and perception‐of‐use factors, and variables that could have affected programme delivery exposure or adherence outcomes. Sampling was done in two areas: Beira district and Dondo district. The sample size calculation (*N* = 600) was driven by assuming a 70% *VitaMais* coverage rate with a ±8.5% relative precision (or a ±6% absolute precision) while assuming a design effect of 2.0 and a 75% response rate. The Mozambique National Institute of Statistics, which has an electronic file consisting of enumeration areas (EA) created for the 2007 national census, selected 40 EA in each stratum (district) where the intervention was being implemented. For Beira district, all 40 EA were selected using population proportion to size (PPS). In Dondo district, 30 EA were initially selected using PPS, and then later, another 10 were selected by PPS. Prior to the next stage of sampling, a household listing was carried out in each selected EA to identify all potentially eligible children 6–23 months. The resulting child lists in each EA were used in the next stage of sampling, which consisted of a simple random selection of 15 children 6–23 months old in each EA. The overall individual response rate was 98.4%, with 99.3% of invited respondents providing questionnaire data in Beira and 97.4% in Dondo. The data are representative of children 6–23 months in Beira district, but we treated the sample in Dondo as a convenience sample as all 40 EA were not selected by PPS at the same time during that stage of sampling. Table 1 shows select demographic characteristics of the survey population.

**Table 1:** Sociodemographic characteristics, household hunger, and infant and young child (IYC) feeding practices for cross‐sectional survey respondents and index child, Mozambique, *n =* 1,028

Characteristic	Beira District (*n* = 481)		Dondo District (*n* = 547)		
	*N* (%)	95% CI	*N* (%)	95% CI	
Sex				
Female	239 (49.7)	[45.2, 54.2]	269 (49.2)	[45.0, 53.4]
Age in months				
6–11	166 (34.5)	[30.4, 38.9]	202 (36.9)	[33.0, 41.1]
12–17	152 (31.6)	[27.6, 35.9]	195 (35.6)	[31.7, 39.8]
18–23	161 (33.5)	[29.4, 37.8]	149 (27.2)	[23.7, 31.1]
Caregiver sociodemographic characteristics				
Respondent relationship to the child				
Mother	452 (94.0)	[91.5, 95.8]	512 (93.6)	[91.2, 95.4]
Age in years					
12–19	75 (15.6)	[12.6, 19.1]	127 (23.2)	[19.9, 26.9]
20–29	295 (61.3)	[56.9, 65.6]	289 (52.8)	[48.6, 57.0]
30–39	81 (16.8)	[13.8, 20.5]	97 (17.7)	[14.8, 21.2]
40+	17 (3.5)	[2.2, 5.6]	23 (4.2)	[2.8, 6.3]
Wealth index^a^				
Low	54 (11.2)	[8.7, 14.4]	151 (27.6)	[24.0, 31.5]
Mid‐low	53 (11.0)	[8.5, 14.1]	153 (27.9)	[24.3, 31.8]
Middle	98 (20.4)	[17.0, 24.2]	108 (19.7)	[16.6, 23.3]
Mid‐high	129 (26.8)	[23.0, 30.9]	77 (14.1)	[11.4, 17.3]
High	147 (30.6)	[26.6, 34.8]	58 (10.6)	[8.3, 13.5]
Household hunger categories^b^				
Little or no hunger in the household	252 (52.4)	[47.9, 56.8]	300 (54.8)	[50.6, 59.0]
Moderate hunger in the household	198 (41.2)	[36.8, 45.6]	222 (40.6)	[36.5, 44.8]
Severe hunger in the household	31 (6.4)	[4.6, 9.0]	25 (4.6)	[3.1, 6.7]
IYC feeding practices				
Key WHO IYC feeding indicators among breastfed children^c^				
Minimum dietary diversity^d^	98 (32.6)	[27.6, 38.2]	114 (27.6)	[23.5, 32.1]
Minimum meal frequency^e^	147 (49.0)	[43.3, 54.7]	202 (48.9)	[44.1, 53.7]
Minimum acceptable diet^f^	64 (21.3)	[17.0, 26.3]	73 (17.6)	[14.3, 21.6]
	(*n* = 301)		(*n* = 414)	
Key WHO IYC feeding indicators among nonbreastfed children^d^				
Minimum dietary diversity^d^	60 (45.8)	[37.4, 54.4]	92 (52.3)	[44.9, 59.6]
Minimum meal frequency^e^	36 (27.5)	[20.5, 35.8]	62 (35.0)	[28.3, 42.4]
Minimum acceptable diet^f^	11 (8.4)	[4.7, 14.6]	22 (12.4)	[8.3, 18.2]
	(*n* = 131)		(*n* = 177)	

*Note*. IYF: infant and young child; WHO: World Health Organization.

a Wealth index such as described by Shea and Johnson (2004).

b Household hunger scale as described by Ballard et al., (2011).

c WHO infant and young child feeding indicators (WHO et al., 2008).

d Proportion of children 6–23 months who receive foods from four or more food groups the previous day. Food groups include: (a) grains, roots and tubers, (b) legumes and nuts, (c) dairy products (milk, yogurt, and cheese), (d) flesh foods (meat, fish, poultry, and liver/organ meats), (d) eggs, (e) vitamin‐A rich fruits and vegetables, and (f) other fruits and vegetables. Diversity scores for breastfed and nonbreastfed children should not be directly compared because breastmilk is not “counted” in any of the above food groups.

e Proportion of breastfed and nonbreastfed children 6–23 months who receive solid, semisolid, or soft foods the minimum number of times or more the previous day. Breastfed children who received solid, semisolid, or soft foods at least twice a day for children 6–8 months and at least three times a day for children 9–23 months and nonbreastfed children 6–23 months who received solid, semisolid, or soft foods or milk feeds at least 4 times a day.

f Proportion of children 6–23 months who receive a minimum acceptable diet (apart from breast milk). This composite indicator defined as breastfed children 6–23 months who had at least the minimum dietary diversity and the minimum meal frequency during the previous day and nonbreastfed children 6–23 months who received at least two milk feedings and had at least the minimum dietary diversity not including milk feeds and the minimum meal frequency during the previous day.

### 2.4 Focused ethnographic study

The FES was conducted during November and December 2016, approximately 11 months. After initiation of MNP distribution to learn from the perspectives of caregivers, programme managers, and programme implementers (i.e., MISAU health centre staff, SC promoters, *activistas*, *Troca Aki* vendors). We used different data collection protocols for each respondent type as described below.

#### 2.4.1 In‐depth interviews with caregivers

Among caregivers, we used in‐depth interviews with open‐ended, guided questions, which were administered with extensive probing to expand and interpret the initial responses. The interview protocol is described in an accompanying paper of this supplement (Schnefke et al., 2019). Interviews were conducted in caregivers' homes, their language preference (Sena, Ndau, or Portuguese), and averaged 2–3 hr each. Three health areas per district were selected based on reported low, medium, and high voucher redemption according to the *Movercado* platform data. Two communities in each of the six health areas, the closest and furthest in distance from the health centre, were chosen to conduct caregiver interviews. Respondents were randomly selected from lists of caregivers of children 6–23 months who had been registered by MISAU or SC as receiving *VitaMais* within 1–3 months prior to the interview date. The sample design required filling respondent categories based on subgroups (age of child and experience with *VitaMais*). We aimed to interview a total of five caregivers in each community: three caregivers (two with a child 6–11 months and one with a child 12–23 months) who reported were currently using *VitaMais* (referred to in this paper as “Continuing Users”); one caregiver of a child 6–23 months who reported to have discontinued using (referred to as “Non‐continuing Users”); and one caregiver of a child 6–23 months who reported not redeeming vouchers (referred to as “Non‐redeemers”). Fifty‐nine of 60 (98.3%) caregivers invited to participate agreed to complete the interview; a single caregiver classified as a non‐redeemer was unable to attend the interview and was not replaced. Table 2 presents the number of respondents per respondent type (as described above) and a summary of selected sociodemographic characteristics of the respondents.

**Table 2:** Respondent type and sociodemographic characteristics of focused ethnographic study (FES) caregiver in‐depth interviews respondents and index child, Mozambique, *n =* 59

Characteristic		Beira District (*n* = 29)	Dondo District (*n* = 30)
Purposeful sampling of respondents			
Respondent type (*n*)	“Continuing Users”^a^	18	18
	“Non‐continuing Users”^b^	6	6
	“Non‐redeemers”^c^	5	6
Child characteristics			
Age of index child (*n*)	6–11 months	14	15
	12–23 months	15	15
Household and caregiver characteristics			
Age of respondent (years)		25.4 ± 9.3 (17–62)^d^	23.1 ± 4.6 (16–39)^d^^,^ e
Is biological mother of index child (*n*, yes)		28	28
Schooling of caregiver (*n*)	Illiterate	0	3
	Primary education (grades 1–8)	16	20
	Secondary education (grades 9–12)	13	7
Household size		5.8 ± 1.9 (3–11)^d^	6.1 ± 1.5 (4–9)^d^

a Caregivers who reported currently using *VitaMais* (received *VitaMais* via vouchers or an alternative pathway and are currently using *VitaMais* or finished their supply).

b Caregivers who reported to have discontinued using (received *VitaMais* via vouchers or an alternative pathway but stopped using it).

c Caregivers who reported not redeeming vouchers (received vouchers but did not redeem them and never received *VitaMais* via an alternative pathway).

d Mean and standard deviation; range in parentheses.

e Missing data for three respondents.

#### 2.4.2 Semistructured interviews with programme managers

Semistructured interviews were completed between November and December 2016 with 17 programme managers as described in Table 3. The protocol contained five modules with open‐ended questions that covered a range of issues including their experiences with and perceptions about designing the trial, training and preparation of programme implementers, functioning of the voucher and *VitaMais* supply chain, programme delivery challenges and successes, and recommendations for how programme delivery could be improved. Knowledge of IYC feeding and *VitaMais* recommendations was assessed through a structured interview with precoded questions. Respondents were purposefully chosen for interviews based on their roles in programme delivery and included central and provincial level programme managers working for SC, PSI, GAIN, and MISAU, high level staff of the District Directorate of Health, and a nutrition technician of the Sofala Provincial Directorate of Health and a GAIN provincial focal point (none of the respondents are authors of this paper).

**Table 3:** Description and sample size of focused ethnographic study participants for the *VitaMais* formative evaluation in Dondo and Beira districts, Mozambique

Programme managers selected for semistructured interviews (*n =* 17)	
Respondent type	Number of respondents
Central level programme managers (GAIN, SC, PSI, MISAU)	4
SC project manager, Beira	2
PSI project manager, Beira	3
District Directorate of Health technical staff	6
Nutrition technician of the Sofala Provincial Directorate of Health	1
GAIN provincial focal point	1

Programme implementers selected for survey (*n =* 168)			
Respondent type	Responsibilities	Beira District (*n =* 103)	Dondo District (*n =* 65)
MISAU health centre staff	Registering and distributing vouchers, providing counselling on MNP and IYC feeding, and conducting informative speeches (*palestras*) and culinary demonstrations. Completing monitoring registration forms. Providing supervision to *activistas*.	15	15
SC promoters	Registering and distributing vouchers, providing counselling on MNP, revisiting caregivers, and conducting informative speeches (*palestras*) and culinary demonstrations. Completing monitoring registration forms. Providing supervision to *activistas*.	27	n/a
MISAU/SC *activistas*	Registering and distributing vouchers, providing counselling on MNP, revisiting caregivers, and conducting informative speeches (*palestras*) and culinary demonstrations. Completing monitoring registration forms.	30	30
*Troca Aki* vendors	Redeeming MNP vouchers (both electronic and paper vouchers) and reporting codes into the *Movercado* monitoring system.	25	14
Community leaders	Mobilization activities such as presence and speeches during theatre performances, radio spots, and other gatherings.	6	6

*Note*. GAIN: Global Alliance for Improved Nutrition; IYC: infant and young child; MISAU: Mozambique Ministry of Health; MNP: micronutrient powders; PSI: Population Services International; SC: Save the Children.

#### 2.4.3 Surveys with programme implementers

A structured interview with precoded questions was conducted between November and December 2016 among 168 programme implementers in Beira (*n =* 103) and Dondo (*n =* 65), as described in Table 3. The respondents included MISAU health centre staff, SC promoters, MISAU/SC *activistas*, *Troca Aki* vendors, and community leaders. All of the programme implementers working in the six health areas selected for the caregiver interviews were invited to participate. One hundred sixty‐eight of 173 (97.1%) agreed to complete the interview; two *Troca Aki* vendors were unable to attend the interview and were not replaced.

##### Quantitative data analysis

Quantitative data were double entered into a customized data entry programme and examined for missing values and outliers. The final dataset was converted to IBM SPSS Statistic, Version 22.0 for descriptive data analysis including frequencies, Pearson chi‐squared continuity correction, Student's *t* test, and Pearson chi‐squared tests. IYC feeding variables were defined according to WHO indicators (WHO et al., 2008); a household hunger scale (Ballard, Coates, Swindale, & Deitcher, 2011) and wealth index (Shea & Johnson, 2004) were calculated. The survey was considered representative of Beira district but not Dondo district, where the data are representative only of the children in each selected EA. Therefore, the analysis did not consider the complex sample design for either district. All children in the selected enumerator areas had an equal probability of selection within the EA, and no weighting was applied.

**Figure 2:**
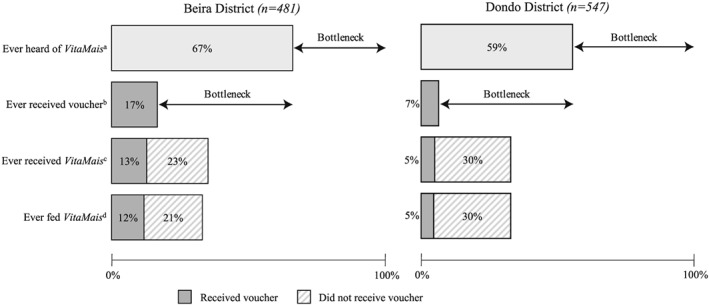
Implementation bottlenecks for *VitaMais* redemption and use pathway among all caregivers by district, Mozambique. ^a^“Ever heard of *VitaMais*,” which is the proportion of caregivers with children 6–23 months who ever heard of *VitaMais*, the brand of micronutrient powder used in the programme, 66.5% (CI [62.2, 70.6]) in Beira District and 59.0% (CI [54.9, 63.1]) in Dondo District. ^b^“Ever received voucher,” which is the proportion caregivers with children 6–23 months who ever received a voucher, 16.8% (CI [13.7, 20.5]) in Beira District and 6.8% (CI [4.9, 9.2]) in Dondo District]. ^c^“Ever received *VitaMais*,” which is the proportion caregivers with children 6–23 months who ever received *VitaMais*, 12.9% (CI [10.2, 16.2]) with voucher and 22.5% (CI [18.9, 26.6]) without voucher in Beira District and 5.3% (CI [3.7, 7.5]) with voucher and 29.6% (CI [25.9, 33.6]) without voucher in Dondo District. ^d^“Ever fed *VitaMais*,” which is the proportion of caregivers with children 6–23 months who ever fed *VitaMais* to the child, 11.9% (CI [9.2, 15.1]) with voucher and 21.4% (CI [18.0, 25.3]) without voucher in Beira District and 4.9% (CI [3.4, 7.1]) with voucher and 30.0% (CI [26.3, 34.0]) without voucher in Dondo District

For the analysis of implementation bottlenecks, we adapted the Tanahashi (1978) model of measuring health service coverage. Using this model, we defined four outcome indicators: three related to handovers from the programme delivery system coverage to the household delivery system and one related to use within the household delivery system as shown in Figure 2. A large difference between adjacent outcomes of the proportion of the target population accomplishing the outcome implies the existence of an implementation bottleneck.

##### Thematic analysis of text

All caregiver and programme manager interviews were recorded, translated to Portuguese and English, and transcribed. University students who spoke fluently the local language and Portuguese were hired to translate and transcribe the recordings. A professional translator was used to translate the Portuguese transcriptions into English. Translations and transcripts were checked twice by different members of the research team. Thematic analysis of text (the transcribed and translated recordings of the caregiver and programme manager interviews) followed basic principles of qualitative text analysis (Miles, Huberman, & Saldaña, 2014). We used QSR International's NVivo Version 11 qualitative data analysis software to organize data and facilitate coding (QSR International, 2002). Data analysis of the caregiver interviews was conducted by a team. First, coding was completed on a sample of transcripts. From those initial transcripts, the team developed a common coding framework. The remaining transcripts were coded by two members of the study team with ongoing input from the other study team members. During weekly study team meetings over the course of 3 months, we adjusted the coding framework, identified emerging themes, and created matrices of quotations that were used to compare themes across transcripts.

## 3 RESULTS

The results of the cross‐sectional survey revealed bottlenecks in the transfer of messages, vouchers, and *VitaMais* between the programme delivery system to caregivers. About one third of caregivers in Beira and about four in 10 caregivers in Dondo had not heard about *VitaMais* (Figure 2). A small proportion of caregivers reported having received a voucher (16.8% in Beira and 6.8% in Dondo), making that step the largest programme implementation bottleneck. However, the survey also revealed that, unexpectedly, many caregivers received *VitaMais* through mechanisms that bypassed the voucher system (22.5% in Beira and 29.6% in Dondo). Virtually, all caregivers who received *VitaMais* reported ever feeding it to their child.

We had assumed that the accessibility of vouchers and *VitaMais* would be improved through *activistas* located in communities where caregivers live, but health centres were the most commonly accessed distribution point (Table 4). Among caregivers who ever heard about *VitaMais*, more than two‐thirds heard about it at a health centre (67.8% in Beira and 74.6% in Dondo) whereas only about one‐tenth from *activistas* in the community (13.4% in Beira and 5.9% in Dondo). If vouchers were received, it was more commonly from programme implementers stationed at health facilities (65.4% in Beira and 73.0% in Dondo) than *activistas* in the community (16.0% in Beira and 2.7% in Dondo). Only about one in four or one in five caregivers had heard of *Troca Aki* shops, and 4.9% in Beira and 21.6% in Dondo needed help redeeming vouchers at the *Troca Aki* shop. Of those interested in using *VitaMais* in the future, most caregivers preferred health facilities (68.8% in Beira and 79.1% in Dondo) when asked about a future location for redeeming vouchers for *VitaMais*, although 30–40% also named shops or the market as potential locations. The majority of caregivers who received *VitaMais* bypassing the voucher system received it at health facilities (54.8% in Beira and 72.2% in Dondo).

**Table 4:** Key aspects for the *VitaMais* programme delivery system for the cross‐sectional survey respondents and index child, Mozambique, *n =* 1,028

Variable	Beira District (*n* = 481)		Dondo District (*n* = 547)	
	*N* (%)	95% CI	*N* (%)	95% CI
Caregiver visited at least once in the last year				
Health facilities	479 (99.6)	[98.3, 99.9]	545 (99.6)	[98.5, 99.6]
*Activistas*	100 (20.8)	[17.4, 24.7]	19 (3.5)	[2.2, 5.4]
	(*n* = 481)		(*n* = 547)	
Where caregivers heard about *VitaMais* from (among those who ever heard of *VitaMais*, multiple options apply)				
Health workers at the health centre	217 (67.8)	[62.5, 72.7]	241 (74.6)	[69.6, 79.1]
*Activistas*	43 (13.4)	[10.1, 17.6]	19 (5.9)	[3.8, 9.0]
*VitaMais* social mobilization events	2 (0.6)	[0.2, 2.5]	15 (4.6)	[2.8, 7.6]
TV and radio	25 (7.8)	[5.3, 11.3]	11 (3.4)	[1.8, 6.0]
Neighbours or other village members	56 (17.5)	[13.7, 22.1]	62 (19.2)	[15.3, 23.9]
Other	31 (9.7)	[6.9, 13.4]	37 (11.4)	[8.4, 15.4]
	(*n* = 320)		(*n* = 323)	
Person from whom the caregiver received the last voucher (among those who received a voucher)				
Health workers at the health centre	29 (35.8)	[26.1, 46.8]	22 (59.5)	[43.2, 73.9]
*Activista* at the health centre	24 (29.6)	[20.7, 40.4]	5 (13.5)	[5.7, 28.6]
*Activista* in the community	13 (16.0)	[9.6, 25.7]	1 (2.7)	[0.4, 16.9]
Through a mobile brigade	5 (6.2)	[2.6, 14.0]	5 (13.5)	[5.7, 28.6]
Other	10 (12.3)	[6.8, 21.5]	4 (10.8)	[4.1, 25.5]
	(*n* = 81)		(*n* = 37)	
Caregiver ever heard of a *Troca Aki* shop				
Yes	132 (27.4)	[23.6, 31.6]	99 (18.1)	[15.1, 21.5]
	(*n* = 481)		(*n* = 547)	
Caregiver needed help redeeming the paper or electronic voucher at a *Troca Aki* shop (among caregivers who received a voucher)				
Yes	4 (4.9)	[1.8, 12.5]	8 (21.6)	[11.0, 37.9]
	(*n* = 81)		(*n* = 37)	
Preferred location to exchange vouchers for *VitaMais* (excluding caregivers who reported not being interested in using *VitaMais* in the future)				
Health centre	297 (68.8)	[64.2, 72.9]	401 (79.1)	[75.3, 82.4]
*Activista* in my community	11 (2.5)	[1.4, 4.5]	9 (1.8)	[0.9, 3.4]
*Troka Aki* shop	15 (3.5)	[2.1, 5.7]	11 (2.2)	[1.2, 3.9]
Other shop	123 (28.4)	[24.4, 32.9]	99 (19.5)	[16.3, 23.2]
Market	47 (10.9)	[8.3, 14.2]	59 (11.6)	[9.1, 14.7]
	(*n* = 432)		(*n* = 507)	
Place of distribution of *VitaMais* without voucher (among caregivers who receive *VitaMais* without redeeming voucher)				
Health facilities	51 (54.8)	[44.6, 64.6]	83 (72.2)	[63.3, 79.6]
*Activistas*	11 (11.8)	[6.7, 20.1]	7 (6.1)	[2.9, 12.2]
Other	31 (33.3)	[24.5, 43.5]	25 (21.7)	[15.1, 30.2]
	(*n* = 93)		(*n* = 115)	

Shifting to the results of the ethnographic study, the FES data revealed the challenges encountered by caregivers with the voucher system and how and why programme implementers bypassed it to deliver *VitaMais*. The results are presented in five themes, which emerged from our exploration of the in‐depth interviews with caregivers, semistructured interviews with programme managers, and surveys with programme implementers.

### 3.1 Theme 1. Caregivers were motivated to redeem vouchers and initiate feeding *VitaMais* to their children because of knowledge of its benefits and authority of health centre staff, SC promoters, and *activistas*

Although *VitaMais* was a new product largely unknown to both programme implementers and caregivers before the programme, caregiver narratives showed that the product was well received, and caregivers were highly motivated to offer it to their children. Among the caregivers interviewed in the FES who initiated feeding *VitaMais* (*n* = 48), the majority (*n* = 40 of 48) reported doing so because of what they learned from programme implementers regarding its benefit(s) for the child, including improving health (*n* = 26), providing vitamins (*n* = 18), improving growth (*n* = 4), improving strength (*n* = 2), and improving appetite (*n* = 1). A theme that emerged from these interviews is the value of vitamins for children's health, as demonstrated in the below quotes:

I give it so that the child is healthy, has vitamin. I can see that my daughter is healthy now, she's having vitamin, she eats eagerly. Continuing User, Dondo

[I give *VitaMais*] to grow well. To grow with good health because *VitaMais* is vitamin and is good for the child … A lot of illness is appearing now but with vitamin, you can cut the reaction to other things. Continuing User, Beira

Among the 48 caregiver respondents, five women attributed their motivation to try *VitaMais* exclusively to the authority of health centre staff or *activistas*, as illustrated in the following quotes:

Because they told me to in the hospital … if it were that I wasn't told in the hospital, if it were that I didn't receive the voucher in the hospital, then I wouldn't have anything to give the child. Continuing User, Dondo

I gave it because they [*activistas*] explained that when you give a child *VitaMais*, the child grows up strong and healthy. Non‐continuing User, Dondo

All of the non‐redeemers (*n* = 11) expressed a desire to feed MNP. Their motivation for wanting *VitaMais* was the knowledge of its benefits for their children, as explained to them by health centre staff, SC promoters, and *activistas*:

[*Activistas* want] children working with them to receive vitamin, for our children to develop well. Non‐redeemer, Dondo

I received the voucher and they [health center staff] told me that I would start to get vitamin for the child … they say that it's good for children with no appetite, the child gets an appetite to eat. Non‐redeemer, Dondo

He [*activista*] gave me the voucher because I wanted to give *VitaMais* for the child because of health … I wanted to but there was work to do here at home so I didn't go and exchange it … I wanted to but I had a lot of work at home and didn't manage to go … You should help me with this voucher. I want them to help me take this voucher and exchange it, and then bring the product home. Non‐redeemer, Dondo

### 3.2 Theme 2. Main obstacles reported by caregivers were not knowing where or how to redeem the voucher or finding *VitaMais* was not available at the shop

Many non‐redeemer caregivers described the challenges they faced in attempting to redeem vouchers starting with poor instructions about where or how to redeem a voucher, as described by one caregiver from Dondo:

*Caregiver* *They have not told me to exchange the voucher yet, so I have not received anything yet. When I received the voucher, I put there on the child's form, so far there's been no result, it is there inside.*

*Interviewer* *When you received the voucher, they did not tell you what to do with it?*

*Caregiver* *Just that I would receive vitamin.*

*Interviewer* *They did not explain that you should go and exchange the voucher and then you would receive the product?*

*Caregiver* *They have not told me yet … They only gave me the voucher.*

Frequently reported obstacles were *VitaMais* stock‐outs or lack of attendants at the *Troca Aki* shop; six of the 11 caregivers interviewed in the FES who did not redeem vouchers described being thwarted by these problems. The following quotes illustrate the determination and frustration of caregivers to redeem vouchers:

I knew what I should do [to redeem the voucher]. I went to the place where I should get the product, I just did not find it, I did not find it, they had already closed. There I stayed without knowing where to take that voucher, ended up not knowing what to do. When I went to the place [*Troca Aki*] to get the product, maybe there I could get all the information of what I was supposed to do afterwards, but when I arrived at the place I did not find anyone. I stayed without knowing where to go. Perhaps if there was somewhere closer it would be easier for me, so I wouldn't have to travel, to look for where I can get it … It's a long way away, a distance. Non‐redeemer, Beira

My daughter took the voucher, went there, arrived there and they said there was no product, it had finished. So, she came back. She went again and they said, come tomorrow. She went again and they said that the product still had not come. She returned and stayed home and that's it. Non‐redeemer, Dondo

When I received the voucher, I was told to go and get the product on the 20th. I went in the evening at 19:00 hours. When I got there, they said there was nothing left and I should return on Friday … I went back at the same time, 19:00 hours. When I arrived there the boy [the *Troca Aki* attendant] contacted [the] health [personnel] to find out if there was a box, the product, and whether he could start to distribute it or not. They agreed and he took out three things and gave them to me, three little boxes. Continuing User, Dondo

### 3.3 Theme 3. Challenges faced by caregivers to redeem vouchers were evident to programme implementers

Approximately half of programme implementers (*n* = 87 of 168 respondents) stated that caregivers encountered difficulties redeeming vouchers. The two most frequently reported barriers included stock‐outs of *VitaMais* (*n* = 54 of 168) and distance to the *Troca Aki* shop (*n* = 33 of 168; data not shown).

These barriers were highlighted by a medical chief respondent who cited distance and time required to seek the *Troca Aki* as reasons why caregivers did not redeem vouchers and the programme did not achieve its objective:

To facilitate a little what I said: the distance, the time … She [caregiver] gets to the health unit and is attended for the first time and the clinical staff and promoters identify the child, register the child in the program, give her the voucher. But in the meantime, the mother must go from the health unit to look for a post [*Troca Aki*] outside … Our objective was not completely achieved, because many mothers held on to the voucher and didn't go to the post.

### 3.4 Theme 4. Health centre staff, SC promoters, *activistas*, and *Troca Aki* attendants helped many caregivers to overcome obstacles to redeem vouchers

Among the caregivers who received *VitaMais* (*n* = 48), only about half obtained it by redeeming a voucher without assistance (*n* = 25 of 48). The others either received a box of *VitaMais* directly from SC promoters or *activistas* (*n* = 14 of 48) or went with SC promoters or *activistas* to redeem the voucher at a *Troca Aki* shop (*n* = 9 of 48). Caregivers reported receiving the product without a voucher from nurses and SC promoters at health centres and *activistas* during home visits. By distributing *VitaMais* directly to caregivers, programme implementers helped caregivers to overcome challenges to redeem vouchers as the following quotes demonstrate:

The activistas came to do this program, but when they arrived I told them “I have the voucher but I haven't yet been to the market to exchange it.” They said “… That's all right … we'll give it to you here so then you can go and give it to the child.” … They took it out and gave it to me. I took the voucher and handed it over. Continuing User, Dondo

I didn't exchange it [the voucher] … My husband prohibited me from going to get it … And so I forgot. And when I went to the hospital on the child's check‐up day, I found that product [*VitaMais*]. I took the product because they didn't require the voucher there. Non‐continuing User, Beira

Half of programme implementers (*n* = 58 of 117) reported finding ways to help caregivers redeem vouchers. The most common solution was that the programme implementer went independently to redeem vouchers at the *Troca Aki* and brought *VitaMais* to the caregivers (*n* = 30 of 58). Programme implementers also redeemed vouchers with caregivers at the *Troca Aki* (*n* = 23 of 58). These findings are supported by *Troca Aki* vendor responses, about two third of *Troca Aki* vendors (*n* = 24 of 39) reported that 1–2 times per week programme implementers took *VitaMais* from the shops to distribute to caregivers directly. Most *Troca Aki* vendors (*n* = 33 of 39) reported being asked about the contents and use of *VitaMais* by caregivers and answering those questions (*n* = 24 of 33), whereas others referred the caregiver to a programme implementer.

### 3.5 Theme 5. Programme implementation was hampered by supply chain challenges

The delivery of *VitaMais* to caregivers required two parallel supply chains, one for vouchers and the other for the product. Paper vouchers were produced by PSI and provided to SC, DDS, and SDSMAS and, in turn, distributed to health workers, SC promoters, and *activistas*. Problems arose from the beginning, starting with incorrect estimations of the number of vouchers needed per population size, which lead to a shortage of vouchers in some areas. About half of health workers, SC promoters, and *activistas* (*n* = 59 of 117) reported not having paper vouchers to distribute to caregivers within 3 months prior to the interview. In most cases, it took weeks (*n* = 24 of 117) or months (*n* = 18 of 117) to receive a new supply of vouchers. Electronic vouchers, delivered to cellphones, alleviated the need for management of physical vouchers, but much fewer were used; according to *VitaMais* tracking redemption data from the *Movercado* platform, 741 electronic and 15,820 paper vouchers in total were redeemed. Both paper and electronic vouchers were affected by difficulties experienced by *Troca Aki* vendors to redeem serial numbers through the mobile phone network (*n* = 16 of 39 reported problems with redemption process due to network issues).

PSI procured and supplied *Troca Aki* shops with *VitaMais*. As observed by caregivers and programme implementers, stock‐outs of *VitaMais* in shops were reported by vendors. Three quarter of vendors interviewed in Beira (*n* = 18 of 25) and half in Dondo (*n* = 7 of 14) reported having had at least one stock‐out of *VitaMais*. The most common reasons were *Troca Aki* vendors not ordering new stock before running out or not being resupplied quickly enough. The time needed to receive new stock varied from 2 or fewer days (24% in Beira and 36% in Dondo) to 2 weeks or more (26% in Beira and 29% in Dondo). Some *Troca Aki* vendors did not understand that redeeming vouchers automatically triggered new stock requests. Instead of continuously redeeming vouchers, several *Troca Aki* vendors reported waiting until the end of the month and redeeming vouchers in a batch.

## 4 DISCUSSION

The intention of the distribution model was to use a market‐based platform that would avoid burdening the public health supply system and increase accessibility of *VitaMais* for caregivers. However, this model required installing two distinct supply chains: one for vouchers and one for *VitaMais*. Consequently, programme implementers and caregivers had to learn about a new product and complex supply, distribution, and redemption processes. The novelty and complexity of the model contributed to programme implementers' difficulty to deliver *VitaMais* and caregivers' hampered efforts to obtain it, which is reflected by the fact that only one third of caregivers received *VitaMais*. The results suggest that caregivers' ability to obtain *VitaMais* was impeded by poor instruction on redemption procedures, extra time required to go to *Troca Aki* shops, and stock‐outs. Fortunately, dedicated and resourceful programme implementers developed strategies to overcome many implementation challenges, primarily through eliminating the need for caregivers to use vouchers. Notably, caregivers reported motivation for redeeming vouchers and using *VitaMais*, and most caregivers who received *VitaMais* fed it to their child. The finding that mothers are motivated to try MNP is consistent with other studies across a range of contexts, for example, Vietnam (Nguyen et al., 2016), Ghana (Aaron et al., 2016), and Ethiopia (Tumilowicz et al., 2019). This suggests that programmes are generally effective in motivating initial use, and more attention is required to improve access to MNP and support continued use.

The results of this formative evaluation can inform MNP programme delivery systems in Mozambique and other contexts more broadly, particularly in terms of the challenges of ensuring that caregivers learn about MNP and easily obtain it. MNP have most commonly been distributed through the health sector free of charge as part of IYC feeding programmes (Reerink et al., 2017). Increasingly fee‐based models are being considered with the intention of lowering intervention costs and building sustainability. Fee‐based models, which reduce or eliminate public sector cost of procuring and distributing MNP, have obvious financial benefits for public health budgets. However, they transfer economic and noneconomic costs to caregivers. To partially mitigate, these costs require careful attention to design and develop efficient and reliable supply chains and convenient distribution locations. The nutrition community can find some models for doing this in other public health sectors, such as reproductive health and malaria prevention where applied marketing principles and techniques have been used to promote public health interventions involving sales and uptake of products (Firestone, Rowe, Modi, & Sievers, 2017).

Caregivers' preference for receiving *VitaMais* at health centres may be the consequence of their difficulty with voucher redemption and inadequate coverage of *activistas*. On the other hand, despite low coverage of *activistas*, for some of the population, they played a key role in obtaining *VitaMais* for caregivers and carrying out BCC activities. In Mozambique, as in many other places, the frontline worker is a minimally paid community member or volunteer. In recent systematic reviews, frontline workers in communities played a central role in ensuring caregivers received MNP (Reerink et al., 2017), and their knowledge and skill were frequently cited as critical for adherence (Tumilowicz et al., 2017). Moreover, MNP distribution through frontline workers in the community compared with health centres may be conditionally more cost‐effective for achieving higher coverage and adherence outcomes, as was recently demonstrated in Uganda (Baker & Vosti, 2018).

Over the last 15 years, MISAU signed codes of conduct for unpaid, volunteer *activistas* with donor agencies and non‐governmental organizations (Ministerio da Saude [MISAU], 2000, 2005) and revitalized its paid community health worker programme (MISAU, 2010). However, both *activistas* and community health workers in Mozambique still face challenges in carrying out their activities (Ndima et al., 2015). Many *activistas* interviewed for this study stated that they faced financial difficulties and requested payment for their services. Considering the low overall voucher and *VitaMais* coverage and ingenuity of *activistas* to overcome barriers with the distribution system demonstrated in this evaluation, there may be a critical role and potential for frontline workers in Mozambique to undertake health and nutrition promotion activities. However, more support is likely required to sustain their work.

Mozambique's public health supply chain frequently suffers stock‐outs of essential medicines (Salomão, Sacarlal, & Gudo, 2017), as do other resource‐constrained systems (Mukasa, Ali, Farron, & Van de Weerdt, 2017). The delivery of *VitaMais* during this short project (15 months) aimed to alleviate the burden of adding another product to this system by using vouchers redeemable through a market‐based platform. Other countries have shown success with similar arrangements to deliver health products, such as insecticide‐treated nets for malaria prevention in Tanzania (Kramer et al., 2017). In addition to overcoming supply chain constraints, vouchers are also a means to subsidize health goods and services (Brody, Bellows, Campbell, & Potts, 2013). Although the *VitaMais* voucher system faced difficulties, voucher programmes have shown promise in other contexts where the investment and willingness to make necessary adjustments based on initial challenges can be ensured. The Tanzania National Voucher Scheme required years and considerable course corrections to programme design to achieve high coverage (Kramer et al., 2017). For example, if further assessment shows that the benefits of not using the public health supply system outweigh the costs of sustaining it, *activistas* could continue to redeem vouchers for caregivers or the role of the *Troca Aki* shop could be changed to supply the health centre rather than the caregiver directly. Programmes need time and resources to apply lessons learned and iterative improvement processes to take place.

In retrospect, we see that the study could have been strengthened by sequencing the cross‐sectional survey and FES rather than conducting them simultaneously. Had the cross‐sectional survey results been available before designing the FES, it would have been possible to more fully explore the population's response to the voucher system and the motivations of programme implementers in their activities to circumvent direct household voucher redemption. For example, we do not have sufficient evidence to more fully explore how demand (in addition to supply) for the product facilitated or inhibited voucher redemption. Conversely, carrying out the FES before the cross‐sectional survey could have allowed for quantitative assessment of the frequency of behaviours and circumstances uncovered in the caregiver narratives. The sampling approach of caregivers interviewed as part of the FES, selecting only caregivers who had received vouchers or *VitaMais*, also limited data collection about why one third of caregivers never heard about the programme.

Using an ethnographic, mixed methods approach in the formative evaluation allowed us to explore unexpected programme outcomes and triangulate findings. Ethnographic methods resulted in key learning about caregiver's attempts to redeem vouchers and how the programme design can be improved. It is still rare in implementation research in nutrition to find evaluations of MNP programmes or international nutrition programmes more broadly, using ethnography to study population responses to interventions (Tumilowicz et al., 2017; Tumilowicz, Neufeld, & Pelto, 2015). We hope this example will encourage others to make use of this powerful tool and the value of mixed methods designs to build knowledge for programme improvement.

## CONFLICTS OF INTEREST

The authors declare that they have no conflicts of interest.

## CONTRIBUTIONS

AT, EP, MEJ, GHP, and LMN designed the study. KK, JV, and EP supervised data collection. AT, MV, KK, JV, GHP, EP, WG, and AN conducted data analysis. All authors participated in the interpretation of results. AT and MV wrote the manuscript. All authors read, revised, and approved the final version submitted for publication.

## References

[mcn12718-bib-0001] Aaron, G. J. , Strutt, N. , Boateng, N. A. , Guevarra, E. , Siling, K. , Norris, A. , … Myatt, M. (2016). Assessing program coverage of two approaches to distributing a complementary feeding supplement to infants and young children in Ghana. PLoS One, 11(10), e0162462 10.1371/journal.pone.0162462 PONE‐D‐16‐11207 [pii] 27755554 PMC5068796

[mcn12718-bib-1000] Baker, E. , & Vosti, S. A. (2018). Changing how we think about cost‐effectiveness of addressing childhood anemia: Findings from Uganda Micronutrient Powders Pilot. Washington, DC: Strengthening Partnerships, Results, and Innovations in Nutrition Globally (SPRING).

[mcn12718-bib-0002] Ballard, T. , Coates, J. , Swindale, A. , & Deitcher, M. (2011). Household hunger scale: Indicator definition and measurement guide. Washington, DC: Food and Nutrition Technical Assistance II Project, FHI 360.

[mcn12718-bib-0003] Brody, C. M. , Bellows, N. , Campbell, M. , & Potts, M. (2013). The impact of vouchers on the use and quality of health care in developing countries: A systematic review. Global Public Health, 8(4), 363–388. 10.1080/17441692.2012.759254 23336251

[mcn12718-bib-0004] Cove, S. , & Pelto, G. H. (2010). Focused ethnographic studies in the WHO programme for the control of acute respiratory infections. Medical Anthropology, 15(4), 409–424. 10.1080/01459740.1994.9966102 8041238

[mcn12718-bib-0005] De‐Regil, L. M. , Suchdev, P. S. , Vist, G. E. , Walleser, S. , & Peña‐Rosas, J. P. (2013). Home fortification of foods with multiple micronutrient powders for health and nutrition in children under two years of age. Evidence‐Based Child Health: A Cochrane Review Journal, 8(1), 112–201. 10.1002/ebch.1895 23878126

[mcn12718-bib-0006] FAO/WHO (2004). Vitamin and mineral requirements in human nutrition. Geneva: Food and Agriculture Organization of the United Nations & World Health Organization.

[mcn12718-bib-0007] Firestone, R. , Rowe, C. J. , Modi, S. N. , & Sievers, D. (2017). The effectiveness of social marketing in global health: A systematic review. Health Policy and Planning, 32(1), 110–124. 10.1093/heapol/czw088 27476502

[mcn12718-bib-0008] Government of the Republic of Mozambique (2010). Multisectorial plan for chronic malnutrition reduction in Mozambique 2011–2014. Maputo, Mozambique.

[mcn12718-bib-0009] Habicht, J.‐P. , & Pelto, G. H. (2012). Multiple micronutrient interventions are efficacious, but research on adequacy, plausibility, and implementation needs attention. The Journal of Nutrition, 142(1), 205S–209S. 10.3945/jn.110.137158 22113873

[mcn12718-bib-0010] Instituto Nacional de Estatística (2013). District statistics [estatísticas distritais]. Maputo, Mozambique: Instituto Nacional de Estatística.

[mcn12718-bib-0011] Kramer, K. , Mandike, R. , Nathan, R. , Mohamed, A. , Lynch, M. , Brown, N. , … Lengeler, C. (2017). Effectiveness and equity of the Tanzania National Voucher Scheme for mosquito nets over 10 years of implementation. Malaria Journal, 16(1), 255 10.1186/s12936-017-1902-0 28619076 PMC5472959

[mcn12718-bib-0012] Miles, M. B. , Huberman, A. M. , & Saldaña, J. (2014). Qualitative data analysis: A methods sourcebook (3rd ed.). Thousand Oaks, Califorinia: AGE Publications, Inc.

[mcn12718-bib-0013] Ministerio da Saude (MISAU) (2000). The Kaya Kwanga commitment—A code of conduct to guide the partnership for health Development in Mozambique. Maputo, Mozambique: Ministério da Saúde Mozambique.

[mcn12718-bib-0014] Ministerio da Saude (MISAU) (2005). Code of conduct governing the partnership between the Ministry of Health and nongovernmental organizations. Maputo, Mozambique: Ministério da Saúde Mozambique.

[mcn12718-bib-0015] Ministerio da Saude (MISAU) (2010). Programa de revitalização dos agentes polivalentes elementares. Maputo, Mozambique: Direcção Nacional de Saúde Pública, Ministério da Saúde Mozambique.

[mcn12718-bib-0016] Ministerio da Saude (MISAU), Instituto Nacional de Estatística (INE) & ICF International (ICFI) (2011). Mozambique demographic and health survey 2011 [Moçambique inquérito demográfico e de saúde 2011]. Calverton, Maryland, USA: MISAU, INE and ICFI.

[mcn12718-bib-0017] Mukasa, B. , Ali, M. , Farron, M. , & Van de Weerdt, R. (2017). Contraception supply chain challenges: A review of evidence from low‐ and middle‐income countries. The European Journal of Contraception & Reproductive Health Care, 22(5), 384–390. 10.1080/13625187.2017.1394453 29087737

[mcn12718-bib-0018] Ndima, S. D. , Sidat, M. , Give, C. , Ormel, H. , Kok, M. C. , & Taegtmeyer, M. (2015). Supervision of community health workers in Mozambique: A qualitative study of factors influencing motivation and programme implementation. Human Resources for Health, 13(1), 63 10.1186/s12960-015-0063-x 26323970 PMC4556309

[mcn12718-bib-0019] Nguyen, M. , Poonawala, A. , Leyvraz, M. , Berger, J. , Schofield, D. , & Nga, T. (2016). A delivery model for home fortification of complementary foods with micronutrient powders: Innovation in the context of Vietnamese health system strengthening. Nutrients, 8(5), 259 10.3390/nu8050259 PMC4882672 27136585

[mcn12718-bib-0020] Pelto, G. H. , Armar‐Klemesu, M. , Siekmann, J. , & Schofield, D. (2013). The focused ethnographic study ‘assessing the behavioral and local market environment for improving the diets of infants and young children 6 to 23 months old’ and its use in three countries. Maternal & Child Nutrition, 9(S1), 35–46. 10.1111/j.1740-8709.2012.00451.x 23167583 PMC6860500

[mcn12718-bib-0021] Pelto, P. J. , & Pelto, G. H. (1978). Anthropological research: The structure of inquiry. New York: Cambridge University Press 10.1017/CBO9780511607776

[mcn12718-bib-0022] QSR International (2002). NVivo. 2.0 edn., Doncaster, VIC, Australia.

[mcn12718-bib-0023] Reerink, I. , Namaste, S. M. L. , Poonawala, A. , Nyhus Dhillon, C. , Aburto, N. , Chaudhery, D. , … Rawat, R. (2017). Experiences and lessons learned for delivery of micronutrient powders interventions. Maternal & Child Nutrition, 13(S1). e12495‐n/a. 10.1111/mcn.12495 PMC5656897 28960878

[mcn12718-bib-0024] Salomão, C. , Sacarlal, J. , & Gudo, E. S. (2017). Assessment of coverage of preventive treatment and insecticide‐treated mosquito nets in pregnant women attending antenatal care services in 11 districts in Mozambique in 2011: The critical role of supply chain. Malaria Journal, 16(1), 223 10.1186/s12936-017-1872-2 28545540 PMC5445451

[mcn12718-bib-0025] Schensul, J. J. , & LeCompte, M. D. (2012). Essential ethnographic methods: A mixed methods approach (2nd ed.). Lanham, Md: Altamira Press.

[mcn12718-bib-0026] Schnefke, C. H. , Tumilowicz, A. , Pelto, G. H. , Gebreyesus, S. H. , Gonzalez, W. , Hrabar, M. , … Vettersand, J. . (2019). Designing an ethnographic interview for evaluation of micronutrient powder trial: Challenges and opportunities for implementation science. Matern Child Nutrition, 15(Suppl. 5), e12804 10.1111/mcn.12804 PMC6856841 31622039

[mcn12718-bib-0027] Shea, R. O. , & Johnson, K. (2004). The DHS wealth index. DHS comparative reports no. 6. Calverton, Maryland: ORC Macro.

[mcn12718-bib-0028] Tanahashi, T. (1978). Health service coverage and its evaluation. Bulletin of the World Health Organization, 56(2), 295–303. 96953 PMC2395571

[mcn12718-bib-0029] Tumilowicz, A. , Habicht, J. P. , Mbuya, M. N. N. , Beal, T. , Ntozini, R. , Rohner, F. , … Neufeld, L. M. (2019). Bottlenecks and predictors of coverage and adherence outcomes for a micronutrient powder program in Ethiopia. Maternal & Child Nutrition. 15(Supp. 5), e12807 10.1111/mcn.12807 31622042 PMC6856804

[mcn12718-bib-0030] Tumilowicz, A. , Neufeld, L. M. , & Pelto, G. H. (2015). Using ethnography in implementation research to improve nutrition interventions in populations. Maternal & Child Nutrition, 11(S3), 55–72. 10.1111/mcn.12246 26778802 PMC5019237

[mcn12718-bib-0031] Tumilowicz, A. , Schnefke, C. H. , Neufeld, L. M. , & Pelto, G. H. (2017). Toward a better understanding of adherence to micronutrient powders: Generating theories to guide program design and evaluation based on a review of published results. Current Developments in Nutrition, 1(6), e001123 10.3945/cdn.117.001123 29955708 PMC5998355

[mcn12718-bib-0032] UNICEF (2013). Community infant and young child feeding (IYCF) counselling package. New York: UNICEF.

[mcn12718-bib-0033] Vossenaar, M. , Tumilowicz, A. , D'Agostino, A. , Bonvecchio, A. , Grajeda, R. , Imanalieva, C. , … Neufeld, L. M. (2017). Experiences and lessons learned for programme improvement of micronutrient powders interventions. Maternal & Child Nutrition, 13(S1). e12496‐n/a. 10.1111/mcn.12496 PMC5656835 28960877

[mcn12718-bib-0034] World Health Organization (2016). WHO guideline: Use of multiple micronutrient powders for point‐of‐use fortification of foods consumed by infants and young children aged 6–23 months and children aged 2–12 years. Geneva: World Health Organization. 28079999

[mcn12718-bib-0035] WHO, UNICEF, USAID, AED, UCDAVIS & IFPRI (2008). Indicators for assessing infant and young child feeding practices—Part I: Definitions. Geneva: World Health Organization.

